# Traditional uses of medicinal plants practiced by the indigenous communities at Mohmand Agency, FATA, Pakistan

**DOI:** 10.1186/s13002-017-0204-5

**Published:** 2018-01-09

**Authors:** Muhammad Abdul Aziz, Muhammad Adnan, Amir Hasan Khan, Abdelaaty Abdelaziz Shahat, Mansour S. Al-Said, Riaz Ullah

**Affiliations:** 1 Department of Botany, Kohat University of Science and Technology, Kohat, Khyber Pakhtunkhwa 26000 Pakistan; 2 Department of Botany, Shaheed Benazir Bhutto University Sheringal, District Dir (Upper), Khyber Pakhtunkhwa Pakistan; 3 Department of Pharmacognosy and Medicinal Aromatic, and Poisonous Plants Research Center, College of Pharmacy King Saud University, Riyadh, 11451 Saudi Arabia; 4 Phytochemistry Department, National Research Centre, 33 El Bohouth st., Dokki, P.O. Box 12622, Giza, Egypt

**Keywords:** Traditional knowledge, Herbal medicines, Use value, Relative frequency of citation

## Abstract

**Background:**

Plant-derived products have an imperative biological role against certain pathogenic organisms and were considered to be a major source of modern drugs. Rural people residing in developing countries are relying on traditional herbal medical system due to their strong believe and minimum access to allopathic medicines. Hence, ethnomedicinal knowledge is useful for the maintenance of community’s based approaches under this medical system. Present study was carried out in an unexplored remote tribal area of Pakistan to investigate and document the existing ethnomedicinal knowledge on local flora.

**Methods:**

Data was collected through semi-structured questionnaires from the community members and local herbalists. Use reports (URs) were counted for each species and analyzed through Linear Regression between the number of URs per family and number of plant species per family.

**Results:**

A total of 64 medicinal plant species were recorded belonging to 60 genera and 41 families. Most frequently used plant families in ethnomedicines were Lamiaceae (8 species) and Asteraceae (7 species). Highest URs were recorded for *Caralluma tuberculata* N.E. Br. (49 URs) being followed by *Thymus serphyllum* L. (49 URs), *Fagonia cretica* L. (47 URs), *Plantago lanceolata* L. (45 URs), *Periploca aphylla* Decne. (44 URs), *Citrullus colocynthis* (L.) Schrad. (44 URs), and *Sideroxylon mascatense* (A.DC.) T.D.Penn. (44 URs). New ethnomedicinal uses were reported for *Boerhaavia elongata* Brandegee and *Fumaria officinalis* L. with confidential level of URs from the study area. Nineteen groups of health conditions were recorded during the course of study being treated with medicinal plants. Maximum number of 30 plant species was used to treat digestive problems. Most widely practiced mode of drugs’ preparation and administration was powder. Leaves (30% plants) were the most frequently used plant parts in the preparation of ethnomedicinal recipes.

**Conclusions:**

Current study is an important addition to the field of ethnomedicines. The study reports important medicinal plants from an area, which has not been investigated previously. Traditional knowledge is restricted to health practitioners and elder community members. This knowledge is at the verge of extinction because younger generation is not taking interest in its learning and preservation process. Hence, there is a dire need to phytochemically and pharmacologically test the investigated taxa for the validation of traditional knowledge.

## Background

Plant resources have remained an integral part of human society throughout history. World Health Organization (WHO) estimated that about 80% of the developing world’s population use traditional herbal medicines [1]. In developing countries, traditional medicines provide a cheap and alternative source for primary health care [2–4] due to lack of modern health facilities, their effectiveness, cultural priorities, and choices [5–7]. In developed nations, usage of traditional herbal medicines is also a fast growing phenomenon. For instance in China, traditional herbal preparations account for 30–50% of the total drug consumption. While at the same time, in countries such as Nigeria, Ghana, Zambia, and Mali, the first choice for 60% children suffering with high malarial fever is herbal medicines. In Ethiopia, about 80% of the population use traditional medicines due to the cultural acceptability of healers and local pharmacopeias, comparatively low cost of traditional medicines and lack of access to modern drugs [8]. The documentation of ancestral knowledge in ethnobotanical surveys may cover the existing gap to discover effective drugs [9].

Pakistan is comprised of various climatic zones with unique biodiversity and consists of 6000 plant species, of which approximately 400–600 species are considered to be medicinally important [10, 11]. In the country, several studies have reported the medicinal uses of plant resources [12–19].The folk knowledge on traditional herbal remedies usually transfer from one generation to another generation through oral way [18–21]. In vertical transfer, chances of elimination of knowledge are going in parallel, which poses a huge threat and need to be addressed for preservation. In the last few decades, a significant trend in scientific and commercial interests has been observed due to the cultural acceptability and economic potency of plant-based herbal products across the country [21, 22]. The country has diverse cultures and a variety of languages spoken predominantly in rural and remote areas. People from rural areas have minimum access to healthcare services, which is one of the main reasons for the utilization of traditional herbal medicines in such cultures [23].

Mohmand Agency is a remote tribal area of Pakistan, which is rich and diversified in important medicinal plants. Modernization and exposure to modern pharmaceuticals have significantly affected the traditional practices in the area. The ethnomedicinal knowledge in the study area is gradually heading towards extinction because the old age community members being the main bearer of this knowledge are passing away and younger generation is not interested to take it. Herbal practitioners in the area have sufficient traditional knowledge, but mostly, they are reluctant to disclose it to other community members. Hence, the current study was planned with the objectives to record the traditional knowledge of study area, preserve it in the form of publish literature, and share it with other communities across the globe.

## Methods

### Ethnographic and socioeconomic background of the study area

**Fig. 1:**
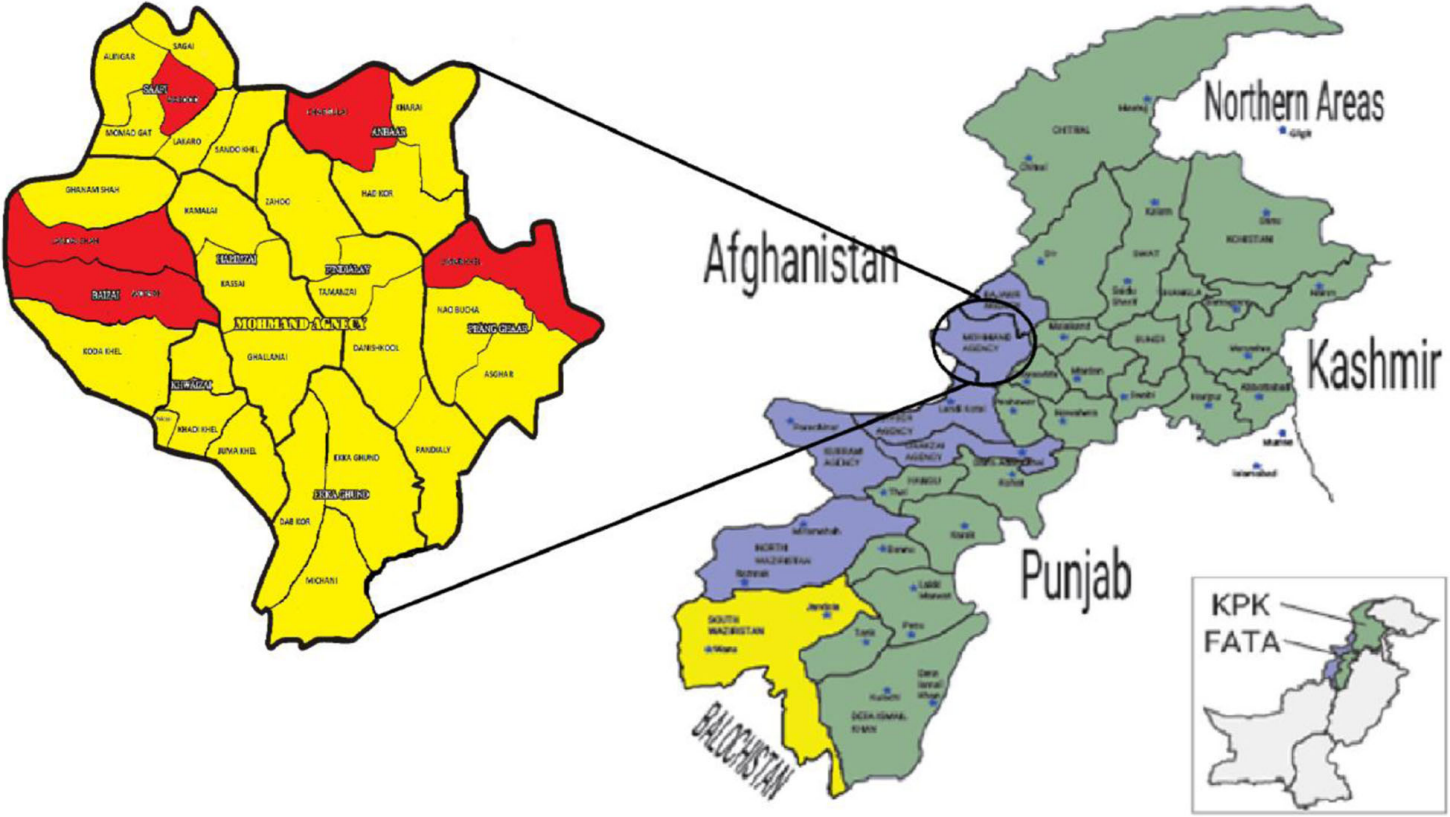
Study area map of Mohmand agency

Mohmand Agency is a part of Federally Administered Tribal Areas (FATA) of Pakistan and established in 1951. The Agency is bordered by Bajaur Agency to the north, Khyber Agency to the south, Malakand and Charsadda districts to the east, Peshawar district to the southeast, and Afghanistan to the west (Fig. 1). Mohmand Agency takes its name from the Mohmand tribe living in the area. Total area of the agency is 2296 km^2^ with headquarter located at Ghalanai area. Geographically, the area is comprised of rugged mountains with barren slopes and widespread along the banks of Kabul River. Lower Mohmand area is rather fertile whereas Upper area is comparatively less productive. Most of the agricultural land is rain fed with insufficient rainfall. Mohmand tribe is also migrated to the fertile lands of district Charsadda and Mardan due to less rainfall ratio and water for irrigation at their homelands. According to the report published by the Pakistan Bureau of Statistics (2017), the current human population of Mohmand Agency is 466,984. Mohmand is the major tribe in the agency which is further subdivided into Tarakzai, Halimzai, Khwaezai, Baezai Safi, and Utmankhel.

The socioeconomic condition of indigenous community is heterogeneous and comparatively poor. The income sources were limited in general except from agriculture and some trade/businesses. Mostly, the people are farmers by profession, while others are government servants, and some have their own small-scale businesses, while some people work on daily wages. Some locals are serving in Gulf States and supporting their families through remittances. People keep domestic animals at their homes, which is a sign of better socioeconomic condition of a tribe or family. There are few secondary schools and only three government colleges in the Agency. There are some public health dispensaries facilitating the people to some extent; however, people residing in remote hilly areas have low or no access to the allopathic medicines. Local communities tend to use traditional herbal therapies as compared to modern pharmaceuticals. They have strong cultural beliefs and faiths about the herbal medicines prepared by the traditional healers locally known *Hakim*(s). Traditional knowledge about the herbal recipe is restricted to these *Hakims* and other elder community members. The socioeconomic background of the indigenous communities can be uplifted if the cultivation and sustainable utilization of medicinal plants is promoted and encouraged in the area.

### Informant selection and ethnomedicinal data collection

Field survey for ethnomedicinal data collection was carried out between May and August 2016. Regular field visits were undertaken prior to data collection in order to ensure and acknowledge the support of the indigenous communities. Local informants were identified for interviews in the month of May, while ethnomedicinal data was recorded in the rest of 3 months. Being local occupant of the study area, Mr. Amir Hasan Khan visited various sites along with volunteer team comprised of a pharmacist and plant taxonomist. The team managed several meetings with the local representatives of the community to whom purpose of the study was presented. Data was collected from community members through semi-structured interviews, meetings, and group discussions at various public places following the procedure adopted by Martin [24]. A total of 81 local key respondents were selected, which include 57 males and 24 females of various age groups through snow ball sampling technique (Table 1). The total respondents also comprised of 14 traditional herbal practitioners locally called *Hakims*. With exception of some elder females, young female community members were not allowed to participate in interviews due to cultural limitations. The selection of respondents was based on their high reputation in ethnomedicinal knowledge. We ensured the validity of the traditional knowledge by maintaining continuous relationships with the local peoples in the course of survey.

**Table 1:** Demographic data of the respondents

Category	Total
Gender	
Man	57
Female	24
Age group	
28–40	8
> 40	73
Education	
Illiterate	45
Primary	9
Middle	12
Secondary	14
Occupation	
House wives	24
Farmers	30
Labors	13
Local healers	14

All interviews with the local people were conducted in local language “Pashto”. In order to get trust and consent, objectives of the study were shared with survey participants. Most of the data on traditional therapies was taken from the local healers. Post data collection, the survey results were redisplayed to the informants for removing errors and omissions from the data.

### Preservation and taxonomical verifications of plant species

Medicinal plants collected during field visits were identified by Dr. Abdul Haq at the Department of Botany, Postgraduate College Khar, Bajaur, Pakistan. The plants were dried under the shade and poisoned with 1% HgCl_2_, pressed and mounted on herbarium sheets. Each herbarium sheet was given a voucher name and number and submitted to the department for future use as a ready reference. Taxonomic problems regarding the correct name and updated systematic position were resolved by using the online database “The Plant List” (www.theplantlist.org).

### Data analysis

Data recorded during the survey was subjected to regression analysis between the URs and number of species per family by using SPSS (16 Version) [25].

## Results and discussion

### Status of the traditional knowledge and role of *Hakim*

Local communities of Mohmand Agency have their own rural culture and beliefs. Their traditional life style including use of herbal medicines make them closer to the natural resources and distinguish from other cultures across the country. It is a natural phenomenon that each community across the globe has a unique philosophy, belief, attitude, culture, and economic status. These are the basic factors, which are responsible for the variation in practicing traditional medicines [26]. The pattern of utilization of medicinal plants in a particular community is a part of its cultural traditional knowledge, passing from one generation to another generation representing a heritage. In the past, several studies have reported the uses of medicinal plants in a single culture or one ethnic group while little attention given to their comparative analysis across various communities and cultures [27]. However, in the last few decades, intercultural importance of medicinal flora has been highlighted among different ethnic groups across the globe [19, 28–32]. This comparisonal approach is practical and essential for finding cross cultural variations and future research prospects on medicinal plants [19].

Current study is an important addition towards the preservation of folk ethnomedicinal knowledge on plants and the efficacy of their derived products from an area not been previously explored. In this study, we have observed that educated people were less conversant compared to the ones with little education in using traditional therapies. Moreover, herbal practitioners hold a large part of the ethnomedicinal knowledge while the aged people only possess a small fraction of this knowledge. In the area, traditional knowledge is under the threat of extinction. The erosion of traditional knowledge is mainly due to the slow and gradual introduction of allopathic medicines, current trend towards modernization, and exposure to technological era. Younger generation is least interested in using herbal therapies; rather, they are more tilted towards allopathic medicines. Similar tendency has been found in other studies [14, 33]. Therefore, the issue of preserving ethnomedicinal knowledge must be properly addressed; otherwise, the vertical and horizontal transfer rate of this knowledge within and across communities would be reduced and ultimately extinct in the near future. This concern has already been elevated in similar studies [34, 35].

Most of the *Hakims* in the study area were using Unani or Ayurvedic system of traditional medication for the treatment of different diseases. Usually, they belong to the local community and have better understandings about the patients’ background, which also facilitates them in disease treatment process. These local herbalists usually diagnose any disease through patient’s symptoms and assessment of the pulse. The *Hakims* interviewed during the study were males, of whom very few were qualified professionals. The local people were of the view that consultation process with the local herbalists mainly depends on personal experiences of these practitioners. However, the introduction of modern pharmaceuticals has triggered the tendency to utilize allopathic medicines and brought cultural changes in the society. Hence, the local dependency on traditional medicines has been significantly decreased as also indicated by Adnan et al. [36].

### Quantitative ethnobotany and preparation of herbal therapies

**Fig. 2:**
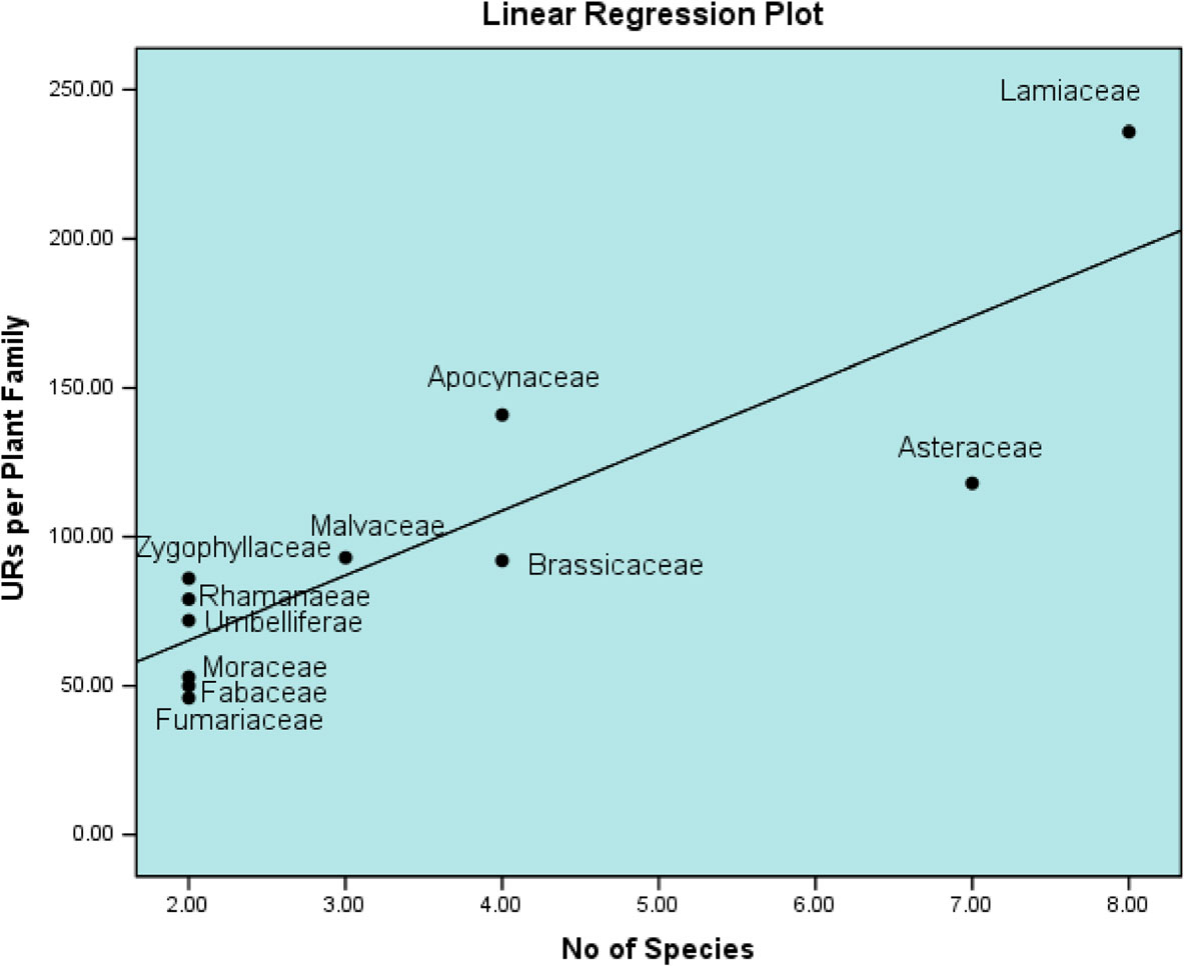
Linear regression between URs per family and number of plant species per family

A total of 64 medicinal plant species belong to 60 genera, and 41 families were catalogued in the study area, which were used for the treatment of several types of human’s diseases (Table 2). Most of the reported plant species belong to the family Lamiaceae (8 species; URs = 236) followed by Asteraceae (7 species; URs = 118), Apocynaceae (4 species; URs = 141), Brassicaceae (4 species; URs = 92), Malvaceae (3 species; URs = 93), Fabaceae (2 species; URs = 50), Fumariaceae (2 species; URs = 46), Moraceae (2 species; URs = 53), Rhamanaeae (2 species; URs = 79), Umbelliferae (2 species; URs = 72), and Zygophyllaceae (2 species; URs = 86). In our study, a significant correlation (*r* = 86) has been observed between the URs and number of species per plant family (Fig. 2). The concept of regression was introduced by Moerman [25] to examine patterns of medicinal plant use, based on taxonomic affiliation. This method includes the following: (i) linearly regress the number of species in a family against the number of medicinal species in the family for a specific geographic region, (ii) interpret least squares line as a measure of average relationship between family size and number of medicinal species, and (iii) use regression residuals to assess medicinal over- or underutilization of groups.

**Table 2:** Medicinal plants used by the indigenous communities in the study area

Family name	Plant name/voucher number	Local names	Habit	Part (s) used	Therapeutic uses	URs
Aizoaceae	*Portulaca oleracea* L./KOH-0057	Warkhari	Herb	Aerial parts	Decoction is made from the aerial part of the plant and is used as demulcent diuretic, vormifuge, laxative and refrigerant.	27
Alliaceae	*Allium cepa* L./KOH-0060	Pyaz	Herb	Bulb	Juice is extracted from the bulb of the herb and is useful in gastric disorders. Also used as diuretic and expectorant.	22
Apocynaceae	*Nerium oleander* L./KOH-0067	Gandiarai	Shrub	Leaves	Leaves decoction is used traditionally for skin diseases and piles. The prepared paste from its root and bark is applied topically after snake bite and scorpion sting. It is given orally for abortion. Leprosy is treated with dermal application of its bark paste.	15
Arecaceae	*Nannorrhops ritchieana* (Griff.) Aitch./KOH-0061	Maizary	Shrub	Leaves	Its leaves decoction is very useful for stomach problems.	41
Apocynaceae	*Caralluma tuberculata* N.E. Br./KOH-0074	Pamankay	Herb	Whole plant	Powder is prepared from the whole plant and given for the treatment of dysentery, jaundice, stomachache, hepatitis (B & C), diabetes and high blood pressure. Also used as carminative.	49
Apocynaceae	*Calotropis procera* (Aiton) Dryand./KOH-0064	Spalmay	Shrub	Leaves	Fever is treated with its leaves’ decoction. Powdered leaves are a useful remedy for wounds, chronic sores and ulcers. Leaves paste is topically applied on inflammation areas (swellings) and rheumatism.	33
Apocynaceae	*Periploca aphylla* Decne./KOH-0087	Barrara	Herb	White latex	The white milk is cure for skin ulcer and wounds. The powder of the plant is used as stomachic and tonic. Also used to treat diarrhea, enlargement of spleen and as diuretic and laxative. Rheumatism is treated with its ash. Whole plant is useful for constipation.	44
Asteraceae	*Xanthium spinosum* L./KOH-0080	Spina ghana	Herb	Leaves, fruits	Leaves and fruits are diaphoretic, diuretic, sedative and used for hydrophobia. Infusion of root is emetic.	14
Asteraceae	*Xanthium strumarium* L./KOH-0059	Mordar botay	Herb	Roots, fruit, seeds	Roots and fruits of the plant are demulcent, stomachic and used in smallpox.	10
Asteraceae	*Artemisia scoparia* Waldst. & Kitam./KOH-0072	Tarkha	Herb	Whole plant	Whole plant infusion is used as purgative.	25
Asteraceae	*Aster trinervius* Roxb. ex Roxb./KOH-0062	–	Herb	Root	Extract of its root is used for cough, cold, fever, pulmonary infections, malaria and hemorrhages.	21
Asteraceae	*Cichorium intybus* L./KOH-0075	Tareezax	Herb	Whole plant	The plant juice is used against jaundice, hepatitis, enlarged spleen and diarrhea. Root decoction has diuretic effect. Decoction obtained from the grinded seed is used against obstructed menstruation and for checking bilious vomiting.	23
Asteraceae	*Erigeron canadensis* L./KOH-0051	Sugar botay	Herb	Whole plant	Plants’ extract is used as diuretic and stimulant.	13
Asteraceae	*Launea nudicaulis* Hook. f./KOH-0066	Shodapai	Herb	Leaves	Poultice of its leaves is topically used for fever.	11
Berberidaceae	*Berberis lyceum* Royle/KOH-0114	Kwary	Shrub	Leaves	Powder of its leaves is used against jaundice. The decoction of its root and bark is used as purgative, blood purifier, febrifuge, and as anti-pyretic. Fruit is eaten for kidney problems.	24
Brassicaceae	*Descurainia sophia* (L.) Webb ex Prantl/KOH-0105	Khashir	Herb	Whole plant	Used for fever.	17
Brassicaceae	*Lepidium draba* L./KOH-0098	Bashka	Herb	Whole plant	Used as stomachic and tonic.	13
Brassicaceae	*Raphanus sativus* L./KOH-0108	Molai	Herb	Leaves	Its leaves are used as diuretic and laxative. Powder of its root is used for jaundice, liver ailment, urinary complaints and for the treatment of piles.	39
Brassicaceae	*Sisymbrium irio* L./KOH-0095	Kharkasai	Herb	Seeds	Powder obtained from its seeds is used as expectorant, febrifuge and stimulant. Seed poultice is used for dermal problems.	23
Caesalpinaceae	*Sophora mollis* (Royle) Baker/KOH-0115	Ghuger	Shrub	Leaves, seeds	Powder of its leaves and seeds are used as anthelmintic.	32
Cannabinaceae	*Cannabis sativa* L./KOH-0109	Bhang	Herb	Aerial parts	Ariel parts’ decoction is used as sedative, analgesic and antispasmodic. Also used for insomnia, depression, neuralgia, asthma and glaucoma. It works also as cooling agent, stimulant, tonic and for the treatment of urinogenital diseases	41
Capparidaceae	*Cleome brachycarpa* (Forssk.) Vahl ex DC./KOH-0103	Zachawa	Herb	Leaves	Its leaves’ paste is used topically against fever.	13
Chenopodiaceae	*Chenopodium album* L./KOH-0112	Sarmy	Herb	Whole plant	Powder made from whole plant is used as/for anthelmintic, jaundice, liver diseases, appetite, diuretic, aphrodisiac, tonic and abdominal pain. Whole plant extract is used for the removal of kidney stone.	31
Convolvulaceae	*Convolvulus arvensis* L./KOH-0100	Parwathiay	Herb	Whole plant	Plant powder is best remedy for skin diseases. Root powder is purgative. Decoction of leaves is used for abnormal menstrual flow. Poultice of leaves is used as antiseptic.	42
Cucurbitaceae	*Citrullus colocynthis* (L.) Schrad./KOH-0113	Marrkonday	Herb	Fruit	Juice of its fruit is used in dropsy. The juice of its fruit is applied to skin problems such as leukoderma. Its oil is best remedy for snake bite. Fruit is purgative and used for cattle intestinal disorder.	44
Cupressaceae	*Cupressus sempervirens* L./KOH-0096	Sabar dana	Tree	Fruit	Its fruits produce cooling effect in cattle.	21
Ephedraceae	*Ephedra intermedia* Schrenk & C.A.Mey./KOH-0106	Mowa	Shrub	Stem	The decoction of stem is used as/for rheumatism, syphilis nasal congestion, bronchial congestion colds cough, flu and asthma.	32
Fabaceae	*Alhagi maurorum* Medik./KOH-0111	Sar azghi	Shrub	Aerial parts	Powder of the plant is used as diaphoretic, expectorant, laxative, anti-diarrheal and antiseptic agent. Root is used in kidney problems.	31
Fabaceae	*Prosopis juliflora* (Sw.) DC./KOH-0071	Kikrye	Shrub	Leaves	Leaves decoction is used for lactation and as expectorant.	19
Fumariaceae	*Fumaria indica* (Hausskn.) Pugsley/KOH-0081	Paparie	Herb	Whole plant	Whole plant powder is diuretic, diaphoretic, aperients and is used for cooling purpose.	28
Fumariaceae	*Fumaria officinalis* L./KOH-0084	–	Herb	Aerial part	It is use in/as blood purification, laxative and skin anti allergy.	18
Geraniaceae	*Geranium wallichianum* D.Don ex Sweet/KOH-0070	Ranjot	Herb	Rhizome	Powder of its rhizome is used against high blood pressure, leucorrhea, as tonic and in rheumatic pain.	13
Juglandaceae	*Juglans regia* L./KOH-0110	Ghoz	Tree	Kernels	Kernel is considered as source of tonic.	36
Lamiaceae	*Mentha spicata* L./KOH-0099	Podina	Herb	Whole plant	Decoction of whole plant is used for cough, flatulence and digestive disorders. Whole plant powder is used as stimulant and carminative. Leaves powder is used as anti-pyretic and for bronchitis.	35
Lamiaceae	*Mentha longifolia* (L.) L./KOH-0056	Ilanai	Herb	Leaves	Leaves’ powder, decoction and extract are considered as carminative, astringent and anti-rheumatic. It is also used for nausea, diarrhea and dysentery.	43
Lamiaceae	*Nepeta cataria* L./KOH-0080	Chemjanbetai	Herb	Leaves	Leaves’ powder is used as carminative, diaphoretic, refrigerant and stimulant. Infusion of its leaves is best for cold and cough. It has also sedative properties.	31
Lamiaceae	*Perowskia atriplicifolia* Benth/KOH-0107	Sansube	Herb	Whole plant	Whole plant produces cooling effect. Extracts of its flower is used against fever.	18
Lamiaceae	*Salvia nubicola* Wall. ex Sweet/KOH-0091	Khaner	Herb	Whole plant	Poultice of the plant part is used topically for the treatment of Gangrene.	17
Lamiaceae	*Stachy parviflora* benth./KOH-0104	Sperghunai	Herb	Stem, leaves	Powder of stem and leaves are used as anthelmintic.	21
Lamiaceae	*Teucrium stocksianum* Boiss./KOH-0055	Kastorai	Herb	Whole plant	Plant extract is used for heart pain.	22
Lamiaceae	*Thymus serphyllum* L.**/**KOH-0082	Mervezei	Herb	Whole plant	Whole plant is used as antispasmodic, carminative, stomachic and anti-diuretic. It regulates the menstrual cycle and improves poor vision. It is also used for the treatment of liver, and as tonic. Seed’s powder is vormifuge.	49
Malvaceae	*Hibiscus trionum* L./KOH-0065	Makhey	Herb	Leaves	Leaves paste is applied to skin eruption. Infusion obtained from its flower is effective against diuretic, itching and skin problems. Leaves powder is stomachic.	33
Malvaceae	*Abelmoschus esculentus* L. Moench/KOH-0088	Bhendai	Herb	Leaves	Poultice of leaves is applied topically for irritating skin. The mucilage of fruit is effective in genitor-urinary system.	24
Malvaceae	*Malva neglecta* Wallr./KOH-0102	Pandirak	Herb	Seeds	Seed powder is used for cough and bladder ulcers.	36
Mimosaceae	*Acacia modesta* Wall./KOH-0089	Palosa	Tree	Gums	Gums of the plant are utilized as tonic, stimulant and as demulcent.	28
Moraceae	*Ficus carica* L./KOH-0079	Inzar	Tree	Fruit	The edible fruits are laxative and beneficial for small pox.	25
Moraceae	*Morus alba* L./KOH-0092	Tooth	Tree	Fruit	Fruits are edible and work as laxative.	28
Myrtaceae	*Eucalyptus globules* Labill./KOH-0101	Safirdad	Tree	Leaves	Decoction of its leaves is used for flu and as anti-diabetic.	24
Nyctaginaceae	*Boerhaavia elongate* Brandegee/KOH-0083	–	Herb	Root, leaves	The leaves’ powder is used for swelling and external body infection. The decoction of roots is used against kidney stone.	31
Oleaceae	*Olea cuspidata* Wall./KOH-0058	QalamiKhono	Tree	Leaves, fruit	Leaves are considered best antiseptic while fruits as tonic.	29
Papilionaceae	*Medicago polymorpha* L./KOH-0090	Shapeshthlary	Herb	Leaves, young shoots	The decoction obtained from the fresh leaves and young shoots is used for the regulation of blood pressure and is carminative.	28
Plantaginaceae	*Plantago lanceolata* L./KOH-0078	Ispaghool	Herb	Leaves	Leaves infusion is used as expectorant, emollient, demulcent. It is also useful for cough and bronchitis. Extracts of its seeds are used as purgative and laxative. The powdered leaves are applied topically on inflamed wounds.	45
Poaceae	*Cymbopogan jwarancusa* (jones) Schult/KOH-0053	Marghkai	Herb	Whole plant	The decoction of the plants is used in typhoid fever.	11
Punicaceae	*Punica granatum* L./KOH-0097	Anar	Tree	Fruit	Its fruit is used as tonic and remove iron deficiency. Powdered bark is used in nasal congestions.	32
Rhamanaeae	*Ziziphus nummularia* (Burm.f.) Wight & Arn./KOH-0063	Karkanra	Tree	Fruit	Fruit is used as carminative, sedative, ulcers, tonic and anti-diabetic.	36
Rhamnaceae	*Zizyphus jujuba* Mill./KOH-0086	Bera	Tree	Fruit	Fruits are effective being laxative and used in constipation.	43
Rosaceae	*Duchesnea indica* (Jacks.) Focke/KOH-0052	Balmangai	Herb	Leaves	Powder of leaves is used as/in diuretic, diarrhea and dysentery.	13
Sapotaceae	*Sideroxylon mascatense* (A.DC.) T.D.Penn./KOH-0094	Gurgura	Tree	Fruit	Fruits are being used as carminative, laxative and tonic. It is also effective in treating urinary tract infections.	44
Solanaceae	*Withania coagulans* (Stocks) Dunal/KOH-0076	Kotilal	Herb	Seed, fruit	Seeds of the plant work in dyspepsia, flatulence and stomachache.	41
Thymelaeaceae	*Daphne mucronata* Royle/KOH-0054	Barrah	Shrub	Bark	Powder is made from its bark and is a useful remedy for toothache.	23
Umbelliferae	*Anethum graveolens* L./KOH-0093	Zanrkay	Herb	Whole plant	Whole plant decoction is used as stimulant and carminative.	31
Umbelliferae	*Coriandrum sativum* L./KOH-0085	Danya	Herb	Fruit	Its decoction is used as/in carminative, stimulant, aphrodisiac, refrigerant, colic pains and bleeding piles.	41
Zygophyllaceae	*Fagonia cretica* L./KOH-0069	Spelaghzia	Herb	Whole plant	Whole plant extract is used for the treatment of diabetes mellitus, blood purification, inflammation and abdominal pain. Juice obtained from it leaves is useful as anthelminthic.	47
Zygophyllaceae	*Tribulis teristris* L.*/*KOH-0073	Azghkay	Herb	Seeds	The powder obtained from its seeds is used against the kidney stone.	39

Our results on most reported plant species from Lamiaceae family are in line with previous studies on various cultures [17, 37–39]. The local importance and acceptance of any plant family may be referred to the presence of active phytochemicals, which may be effective in certain pathological conditions [40]. Additionally, the reason behind the usage of a specific family may be due its predominance in a geographical area as well as familiarity among the local people.

**Fig. 3:**
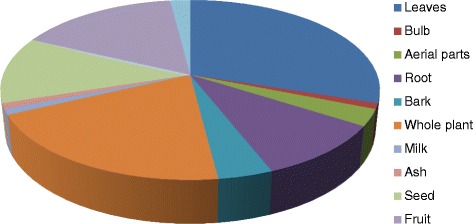
Most frequently used plant parts in the study region

Out of the reported 64 medicinal plants, herbaceous (68%) life form was dominantly used in drug preparation. The most frequent plant parts were leaves (30%) followed by whole plants (20%) and fruits (16%) (Fig. 3). Herbaceous life form and leaves’ usage in ethnomedicinal recipes have been reported in several studies [12, 41–43]. The leaves and aerial plant parts are active in the process of autotrophy and metabolism and can be easily collected [44–47]. However, in Traditional Chinese Medicines (TCM), roots have been indicated as the dominant part in recipes preparation [48, 49]. The composition of a particular ethnomedicine varies from species to species as for one species the active part could be the leaf while for other it may be root. In any case, phytopharmacological screening of all plant parts is necessary to validate the local traditional knowledge and search new compounds for the modern allopathic medicines. In this study, various methods of drug preparation and administration have been documented, which were being applied by the local herbalists. Mainly, the ethnomedicines were administered orally along with other additives. Our results are in line with other studies, in which ethnomedicines were utilized along with some solvents/additives to reduce the bitter taste of the remedy, mitigate the toxic consequences such as vomiting and diarrhea, and maximize drugs’ efficiency [17, 50, 51].

**Fig. 4:**
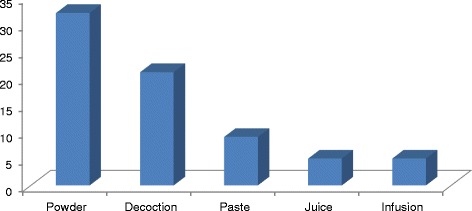
Preparation methods of herbal medicines

In terms of oral use of herbal recipes, mainly, the plant powder was ingested with water or as decoction and very rarely juice was extracted (Fig. 4). These findings are similar to the previous studies [13, 52]. On the other side, paste and grinded herbs were extensively used to treat a particular dermal disease. Other studies have also reported the decoction as the widely accepted administrative form of herbal medicines [17, 41, 53]. Reported medicinal plants were used for various health conditions and diseases. Most of the remedies were based on single plant’s application due to palatability, non-toxicity and high efficacy [41]. Some remedies were prepared in a combination of two or more plants to gain maximum therapeutic effect (synergism).

**Fig. 5:**
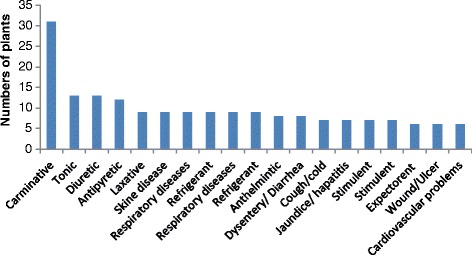
Number of plants used to treat different ailments in the study area

A total of 19 groups of health conditions were recorded based on symptoms (Fig. 5). The local herbalists usually diagnose a specific ailment by symptoms and signs, while not using the modern laboratory techniques. Highest number of plants were used for digestive problems (30 species) followed by as tonic (13 species) and diuretic (13 specie) (Fig. 5). These results are in parallel with the previous findings reported from various parts of the country [18, 19, 36, 54–56], in which gastrointestinal complaints were declared common. The existence of digestive disorders as a main use category in the study area may be due to the ingestion of contaminated foods and other toxic explosive material produced as a result of previous armed conflicts in the area. Furthermore, lack of proper sanitation, less access to clean water, and fuel wood’s smoke inhalation may contribute to gastric problems. Gastrointestinal disorders are predominant across the globe, for which a large number of medicinal plants are being used by different cultures [27, 46, 47, 49, 52, 57–60]. In the study area, local people are aware of the toxic consequences of some orally used medicinal plants such as *Nerium oleander* L. and *Calotropis procera* (Aiton) Dryand. These plants can cause nausea and vomiting in humans and death of cattle if not properly administered.

### Important medicinal plants

Traditional knowledge of medicinal plants has contributed to the modern day pharmaceutics in the form of important drugs. As an example, these include quinine (*Cinchona succirubra*), colchicines (*Colchicum autumnale*), digitalis glycosides (*Digitalis* spp.), morphine, codeine, papaverine (*Papaver somniferum*), physostigmine (*Physostigma venenosum*), and pilocarpine (*Pilocarpus jaborandi*) [61]. Hence, the need for searching new products from medicinal plants is essential component for the current and future generations.

In our study, the importance of a medicinal plant species was indicated by use reports (URs). Maximum URs were recorded for *Caralluma tuberculata* N.E. Br. (49), *Thymus serphyllum* L. (49), *Fagonia cretica* L. (47), and *Plantago lanceolata* L. (45). Other plants are important for the indigenous communities; however, they were reported with lower URs. Moreover, new uses of *Boerhaavia elongata* Brandegee and *Fumaria officinalis* L have also been recorded in this study. These species are being discussed as follow:

#### *Caralluma tuberculata* N.E. Br.

*C. tuberculata* locally known as *Pamankay* is extensively used against jaundice, dysentery, stomach pain, high blood pressure and as carminative in the area. In addition, the plant is also being utilized as vegetable and carry high price (4 USD/kg) in the local market. In Pakistan, wild and cultivated *C. tuberculata* is traditionally used in tea for the treatment of diabetes [62].

In Quetta (Pakistan), there is a tradition for the treatment of high blood pressure by chewing fresh plant of *C. tuberculata* after each meal, thrice a day for 1 month [62]. The plant is also utilized for blood purification in South Africa, Saudi Arabia, and Iran [62–64]. Reports have shown its uses for the treatment of digestive disorders such as diarrhea, ulcer, constipation, and abdominal pain [63, 65–67]. Skin problems are also being treated by the use of this plant in Pakistan, India, Nigeria, Iran, Saudi Arabia, and Oman [62, 64, 68]. Furthermore, chewing of fresh plant is considered effective in pimples, freckles, blood purification, rheumatism, and pyrexia [67, 69–71]. *Caralluma* extracts cause the secretion of synovial fluids, which enhances joints mobility and efficiency. The aerial parts of the plant have been scientifically validated for anti-malarial activity [70].

Khan et al. [14] reported that the methanolic extract of *C. tuberculata* has inhibited the growth of *Aspergillus flavus* and *Aspergillus niger*. Parekh and Chanda [72] have also found its antifungal activity against *Pheretima posthuma* and *Candida albicans*. Moreover, chloroform and methanolic extracts of *C. tuberculata* have shown antioxidant activity [73]. Its ethyl acetate extract was found to be the most potent anti-proliferative fraction against breast cancer and other tumor cell lines while the steroidal glycosides were found to possess moderate micromolar cytotoxic activity on breast cancer and other cells [74]. Ethanolic and aqueous extracts of *C. tuberculata* have shown hypoglycemic activity at a dose of 70.42 mg/kg in allaxon-fed diabetic male Albino rats [75]. Various research studies have indicated that the bioactivities of *C. tuberculata* might be due to the presence of certain classes of compounds including pregnane glycosides, flavonoid, flavones, and glycosides [36].

#### *Thymus serphyllum* L.

*T. serphyllum* is widely used as antispasmodic, carminative, stomachic, tonic, and anti-diuretic in the study area. It regulates the menstrual cycle, improves the poor vision, and is used for the treatment of liver disorders. Studies conducted in different parts of the world have shown that the aerial parts of *T. serpyllum* has a long tradition in Europe [76] and worldwide as anthelmintic, antiseptic, antispasmodic, carminative, deodorant, diaphoretic, disinfectant, expectorant, sedative, and tonic [77]. It is most frequently used for gastric problems and respiratory problems [78, 79]. In Western Balkans, the species is used as a sedative [80], improving blood circulation, anticholesterolemic and immunostimulant [81]. In the alpine region of northeastern Italy, infusion or decoction of plant’s aerial parts is used for the treatment of rheumatism [82]. Gairola et al. [32] mentioned the use of wild thyme in some regions of India for the treatment of menstrual disorders while Shinwari and Gilani [83] confirmed its use as an anthelmintic in the Northern Pakistan. *T. serpyllum* is also used externally as an antiseptic and wound-healing agent [84–86].

Over the last two decades, attention has been given to investigate the chemical composition of *T. serpyllum*’s essential oil [87–91]. According to the Physicians’ Desk Reference (PDR) for Herbal Medicines, the chief component of essential oil is carvacrol, while it also contains borneol, isobutyl acetate, caryophyllene,1,8-cineole, citral, citronellal, citronellol, *p*-cymene, geraniol, linalool, *α*-pinene, *γ*-terpinene, *α*-terpineol, terpinyl acetate, and thymol in relatively high concentrations [92]. According to European Pharmacopeia, *T. serpyllum* contain at least 1.2% essential oil, out of which the total content of carvacrol and thymol is 40% or higher [93]. In addition to the essential oil, wild thyme also contains flavonoids, phenol carboxylic acids and their derivatives, triterpenes and tannins [92, 94]. Kulisic et al. [95] also reported *γ*-terpinene and p-cymene among the main components of the essential oil. The compositions and concentrations of compounds in the essential oil of *T. serpyllum* are significantly different across Pakistan and worldwide. For instance, the essential oil of *T. serpyllum* growing in Pakistan contains mainly thymol (53.3%) and carvacrol (10.4%) [88], while another study by Hussain et al. [89] reported carvacrol (44.4%) and ocymene (14.0%) from the Gilgit valley of Pakistan. Hazzit et al. [96] found that the antioxidant potential of *T. serpyllum* may be attributed to the phenol constituents of essential oil, which justifies the traditional uses of wild thyme. However, the antioxidant activity of its essential oil is not only due to the presence of certain dominant components but also the synergism of a larger number of compounds in small amounts including trans*-*nerolidol, germacrene D, *β*-cadinene, and *δ*-bisabolene [26].

Antimicrobial assays revealed that ethanol and aqueous extracts of *T. serpyllum* carries inhibitory activity against *Staphylococcus aureus*, *Bacillus subtilis*, *Escherichia coli*, *Pseudomonas aeruginosa, Proteus mirabilis, Salmonella choleraesuis*, *Enterococcus faecalis, Salmonella Typhi*, *Shigella ferarie*, *Bacillus megaterium*, *Bacillus subtilis*, *Lactobacillus acidophilus*, *Micrococcus luteus*, *Staphylococcus albus*, and *Vibrio cholera* [88, 97, 98]. The hexane extract of the species demonstrated best anticancer activity against HepG_2_ (Liver Carcinoma Cell Line) followed by HCT-116 (Colon Cancer Cell Line), MCF-7 and MDA-MB-231 (Breast Cancer Cell Lines), PC3 (Prostate Cancer Cell Line), and A549 (Lung Carcinoma Cell Line) [99].

#### *Fagonia cretica* L.

In our study, extract from the whole plant of *F. cretica* was utilized for curing diabetes mellitus, blood purification, as anti-inflammatory and for abdominal pain. The juice obtained from its leaves is used as anthelminthic. Aziz et al. [18] reported the extracts of *F. cretica* for the treatment of diabetes mellitus, scabies, gastric problems, expulsion of abdomen worms, blood purification and inflammation. The list of diseases treated by the *F. cretica* includes sore mouth and small pox [100]. Additionally, it is also used as hematological, neurological, endocrinological, dermatological, and anti-inflammatory, for small pox and endothermic reactions in the body [101, 102]; for cold and cough [103]; as astringent, febrifuge, thirst, vomiting, dysentery, asthma, urinary discharges, typhoid, toothache, stomach troubles and anti-tumor [104]. Similarities in plants’ usage in the current study with that of previous studies may be due to similarities in floral composition, propinquity and other cultural values.

Pharmacological studies have shown that *F. cretica* carries anticancer, antimicrobial, antiviral, analgesic, anti-inflammatory, antipyretic, antioxidant and thrombolytic activities [105]. Aqueous extract of *F. cretica* has anti-breast cancer effect without common side effects of standard cytotoxic therapy [106]. Methanolic extract has been reported as hemorrhagic inhibitor against snake venom as compared to standard antiserum [107]. The alcoholic extract of the plant exhibits significant inhibitory potential against *Salmonella typhi* a causative agent of typhoid fever [108]. Moreover, it has also shown inhibitory potential against *Bacillus subtilis, Escherichia coli, Pseudomonas aeruginosa, Staphylococcus aureus*, *Staphylococcus epidermidis*, and *Klebsiella pneumoni* [109, 110]. Phytochemical investigation of the plant revealed the presence of alkaloids, flavonoids, terpenoids, saponins, tannins, coumarins, sterols, and glycosides in different polar and non-polar extracts of its parts [109–111]. Anjum et al. [112] isolated 11 new compounds from the *n*-hexane extract of *F. cretica* including Linoleic acid, b-sitosteryl-3-O-b-D-(60-hexadecanoyl)-glucopyranoside, octacosonic acid, methyl triacantanoate, b-Amyrin acetate, taraxerol, oleanolic aldehyde acetate, triacontanoic acid, taraxerone,2a,3a,23 trihydroxyolean-12-en-28-oic acid, 3a,23-dihydroxyrus-12-en-28-oicacid. Isolated compounds from *F. cretica* extracts indicated antifungal potential against *Trichophyton longifusus, Candida albicans, Aspergillus flavus, Microsporum canis, Fusarium solani*, and *Candida glabrata* strains. Among 11 isolated compounds, “taraxerol” has shown highest inhibitory effects against *Aspergillus flavus* with 90-mm zone of inhibition compared to the 20 mm for Miconazole standard [112]. The pharmacological evidences of this rare plant in terms of antimicrobial, anticancer, and other activities suggest further clinical trials to validate its traditional uses.

#### *Plantago lanceolata* L.

Infusion obtained from the leaves of *P. lanceolata* is used as expectorant, emollient and demulcent in the study area. It is useful for cough, cold, fever and bronchitis while the extract is used as purgative and laxative. The powder obtained from the leaves is applied topically on inflamed wounds. In previous studies, *P. lanceolata* has been reported for the treatment of cough, bronchitis, and as stomachache, dysmenorrheal, expectorant, emollient, demulcent, astringent and laxative [18, 54, 79]. Phytochemical constituents isolated from this plant include silica, potassium, alphaamyrin, mucilage, zinc, glycosides, caffeic and tannins. Khalid et al. [113] revealed that *Plantago* has demulcent, astringent expectorant, having healing and soothing effect on intestinal mucosal layer. Reports have shown that the crude extract of *P. lanceolata* have the potential to combat with multidrug resistant *K. pneumonia* [114, 115].

#### *Boerhaavia elongata* Brandegee and *Fumaria officinalis* L.

Powdered leaves of *B. elongate* are used for swellings and external body infections while the decoction of its root is used against kidney stones. In the same way, *F. officinalis* is utilized for the cure of blood purification, skin problems, and allergy and as laxative. A detailed and comprehensive literature survey was carried out by investigating various bibliographic sources in order to sort out the novelty of the reported indigenous flora [12–18, 22, 42, 43, 54, 83, 88, 115–125]. After thorough search, no previous reports were found for the two medicinal plants. However, new medicinal uses were found with moderate number of URs from the study area for *B. elongata* and *F. officinalis*, which are scarcely mentioned in previous ethnobotanical studies across the country. Other important plants, which were commonly used by the local community having high URs, were *Mentha longifolia* (L.) L. (43), *Zizyphus jujuba* Mill. (43), *Nannorrhops ritchieana* (Griff.) Aitch. (41), *Cannabis sativa* L. (41), *Withania coagulans* (Stocks) Dunal (41), and *Coriandrum sativum* L. (41). These species require comprehensive phytopharmacological investigations to validate their efficacy and ensure their safe utilization.

### Threats to the indigenous flora

During the field survey, certain important plants were found under the threat of anthrpogenic pressure. As an example, *C. tuberculata* and *N. ritchieana* were found endangered in their natural habitats due to over collection. These are the two species, which are harvested by the local people for economic benefits. The local people claimed that fuel wood collection, roads and homes construction, uncontrolled fire setting, fodder collection, and over-grazing are the possible factors responsible for the destruction of the natural habitats of the medicinal plants. The rate of such activities varies from place to place. People are unaware of the conservation of the medicinal plants. In addition to the aforementioned threats and processes, armed conflict in the area for the last one decade is an alarming concern for the conservation of the medicinal flora. The use of explosive material in the area has made several plants contaminated with corrosive material. Furthermore, no conservation strategies have been adopted in the study area to avoid overexploitation of the wild species. However, some plants are being cultivated and marketed by the farmers, which include *Morus alba*, *Olea cuspidata*, and *Punica granatum.* This local initiative of the indigenous communities to gain economic benefits from the local flora may promote the interest for the conservation and regulation of local flora to safeguard the threatened species [126].

## Conclusions

This study has played an important role in the preservation of traditional knowledge from a remote area, where the folk knowledge is eroding at a faster rate due to several factors including the rapid modernization. The traditional knowledge is mostly in the custody of local herbalists and elder community members. The study has reported a total of 64 medicinal plant species belonging to 36 families. Lamiaceae and Asteraceae were the utmost used plant families in the study area. Species such as *C. tuberculata* and *T. serphyllum* have highest number of use reports and are mostly used by the local people. Two medicinal plant species including *B. elongata* and *F. officinalis* were reported with new ethnomedicinal uses with confidential level of citations from the study area. Certain medicinal plants have reportedly been screened phytochemically and tested pharmacologically; however, the traditional uses of a large number of plants still remain to be validated. Hence, our study stress on the need for the phytochemical, pharmacological, microbiological, toxicological, preclinical, and clinical investigations to ensure the safety and efficacy of the reported medicinal taxa. Our study also highlighted certain threats faced to the local flora including deforestation, heavy grazing, and overexploitation that are affecting the process of sustainability. Hence, sound conservation strategies need to be developed and implemented for the sustainable utilization of medicinal flora and preservation of traditional knowledge.

## Abbreviations

FATA: Federally Administered Tribal Areas
TCM: Traditional Chinese Medicines
URs: Use reports
WHO: World Health Organization
